# Multiparametric CMR imaging of myocardial structure and function changes in diabetic mini-pigs with preserved LV function: a preliminary study

**DOI:** 10.1186/s12872-022-02597-7

**Published:** 2022-04-02

**Authors:** Guozhu Shao, Yukun Cao, Yue Cui, Xiaoyu Han, Jia Liu, Yumin Li, Na Li, Heshui Shi

**Affiliations:** 1 Department of Radiology, Union Hospital, Tongji Medical College, Huazhong University of Science and Technology, Wuhan, 430022 People’s Republic of China; 2 Hubei Province Key Laboratory of Molecular Imaging, Wuhan, 430022 People’s Republic of China

**Keywords:** Diabetes mellius, Cardiac magnetic resonance, Epicardial adipose tissue, Feature tracking

## Abstract

**Background:**

The purpose of this study is to dynamically monitor the myocardial structure and function changes in diabetic mini-pigs by 1.5 T cardiac magnetic resonance.

**Methods:**

Three male mini-pigs underwent cardiac magnet resonance (CMR) imaging, and histologic examination. T1-mapping was acquired at basal, mid and apical segments. CMR feature-tracking (CMR-FT) is used to quantify left ventricle global longitudinal (LVGLS), circumferential (LVGCS) and radial strain (LVGRS). Epicardial adipose tissue (EAT) was evaluated using a commercially available software.

**Results:**

Left ventricular mass (LVM), myocardial native T1 value, extracellular volume (ECV) value and EAT were increased gradually after 6 months of modeling, while LVGLS decreased gradually after 6 months of modeling (LVM: 24.5 (23.4, 26.7) vs. 42.7 (41.4, 44.6) g/m^2^, *p* < 0.001; Native T1: 1005.5 (992.6, 1010.7) vs. 1028.7 (1015.5, 1035.6) ms, *p* = 0.041; EAT: 16.1 (14.5, 18.2) vs. 24.6 (20.8, 26.9) mL, *p* = 0.020; ECV: 21.4 (20.2, 23.9) vs. 28.9 (26.7, 30.3) %, *p* = 0.011; LVGLS: − 22.8 (− 21.4, − 23.9) vs. − 17.4 (− 17.2, − 19.2)%, *p* = 0.008). The diffuse myocardial interstitial fibrosis was found in histology samples.

**Conclusion:**

The progressive impairments in LV structure and myocardial deformation occurs in diabetic mini-pigs. T1 mapping and CMR-FT technology are promising to monitor abnormal changes of diabetic myocardium in the early stage of diabetic cardiomyopathy.

## Background

In 2010, the prevalence of diabetes among adults (20–79 years old) was 6.4%, reaching 285 million people, but the prevalence rate will increase to 7.7%, and the number will reach 439 million in 2030 [1]. Diabetes mellitus (DM) is an important risk factor for the morbidity and mortality of cardiovascular diseases, which seriously endangers human health and aggravates the social burden. Diabetic cardiomyopathy (DCM) is a specific myocardial disease that excludes hypertension or coronary artery disease and causes cardiac structural and functional abnormalities in patients with long-term insulin resistance or hyperglycemia [2, 3]. Many pathological mechanisms promote apoptosis, necrosis and diffuse fibrosis of diabetic cardiomyocytes, and eventually lead to cardiac dysfunction and heart failure [4]. Epicardial adipose tissue (EAT) also plays a particularly important role in diabetes mellitus and cardiovascular physiology. EAT excretes several pro-inflammatory chemokines and cytokines, collectively called adipokines, been shown to impair cardiomyocyte contractile function and fat oxidation [5]. Therefore, early detection of myocardial fibrosis, cardiac dysfunction and EAT and its effective intervention is very important for the prevention and treatment of diabetic cardiomyopathy.

Cardiac magnetic resonance (CMR) cine imaging is the gold standard for evaluating myocardial structure and function in many cardiac diseases. In recent years, as a new and sensitive technique of measuring myocardial deformation and function, CMR tissue tracking (CMR-FT) method based on the cine images has been widely used in myocardial strain analysis of different types of cardiomyopathy [6, 7], and it can also provide important value for prognosis analysis of cardiomyopathy [8]. Besides, CMR T1/extracellular volume (ECV) mapping techniques are of great importance in the diagnosis and treatment evaluation of various heart diseases for their non-invasive early quantitative and monitoring of myocardial focal and diffuse lesions.

So far, the researches on diabetic cardiomyopathy mainly focus on patients with type 2 diabetes [9]. However, few studies have been done on animals with type 1.5 diabetes model (a combination of type 1 and type 2 DM). In this study, we use mini-pigs to simulate type 1.5 DM model and to evaluate its influence on myocardial structure and function by using a variety of advanced CMR quantitative techniques.

## Methods

### Experimental animals

All animal experiments were approved by Institutional Animal Care and Use Committee, Tongji Medical College, Huazhong University of Science and Technology, Wuhan, China and were executed conforming to the “Guide for the Care and Use of Laboratory Animals” and in compliance with the ARRIVE guidelines. Three male mini-pigs, aged 5–6 months, were provided by the Laboratory Animal Center of Tongji Medical College. Type 1.5 DM animal models were induced by intravenous injection of streptozotocin at a dose of 150 mg/kg [10]. Fasting blood glucose was continuously measured with a glycemeter at pre-determined time points. Sustaining fasting blood glucose levels > 250 mg/dL were considered as having diabetes [11]. Their weight ranged from 20 to 24 kg at the beginning and increased to 28–30 kg at the end of this study. At 6 months, after non-invasive imaging the pigs were anaesthesia through intravenous injection of overdoses of 3% pentobarbital sodium (6 mg/kg). The pigs were sacrificed under unconscious (no pedal reflex and no blink reflex, no response to painful stimuli) using the blood-letting method for histologic analyses.

### CMR scanning protocol

The mini-pigs were anesthetized through intravenous injection of pentobarbital sodium (0.1 mg/kg min). All three pigs have a pre-DM scan in our study, and all pigs have a post-DM scan at 1.5, 3, 4.5 and 6 months. Hematocrit level (HCT) was measured by collecting blood from ear vein before MR scanning was performed.

All three pigs underwent a standard CMR examination with a 1.5 T scanner (MAGNETOM Aera, Siemens Healthcare, Erlangen, Germany). A balanced steady-state free procession (b-SSFP) sequence was performed to acquire left ventricular long-axis and short-axis (coverage from the base to the apex segment) cines. The parameters included: repetition time, 2.93 ms; echo time, 1.16 ms; flip angle, 80°; slice thickness, 6 mm; field of view, 340 × 255 mm^2^; and matrix, 256 × 205. T1 mapping technique was performed at the basal, mid and apical slices of the left ventricular short axis before and 15 min after the administration of gadolinium-diethylenetriamine pentaacetic acid (DTPA) (0.2 mmol/kg, Magnevist; Bayer Healthcare; Germany) using a prototype-modifed Look-Locker inversion recovery (MOLLI) sequence. The following parameters were used: repetition time, 3.89 ms; echo time, 1.12 ms; flip angle, 35°; slice thickness, 8 mm; field of view, 360 × 270 mm^2^; matrix, 256 × 192; iPAT factor, 2; and acquisition scheme, 5b(3b)3b. The ECV mapping image is generated automatically by inputting the value of HCT through a prototype inline processing function from Siemens, based on native and post-contrast T1 mapping at the same slice and the subject-specific HCT value.

### MR data analysis

**Fig. 1:**
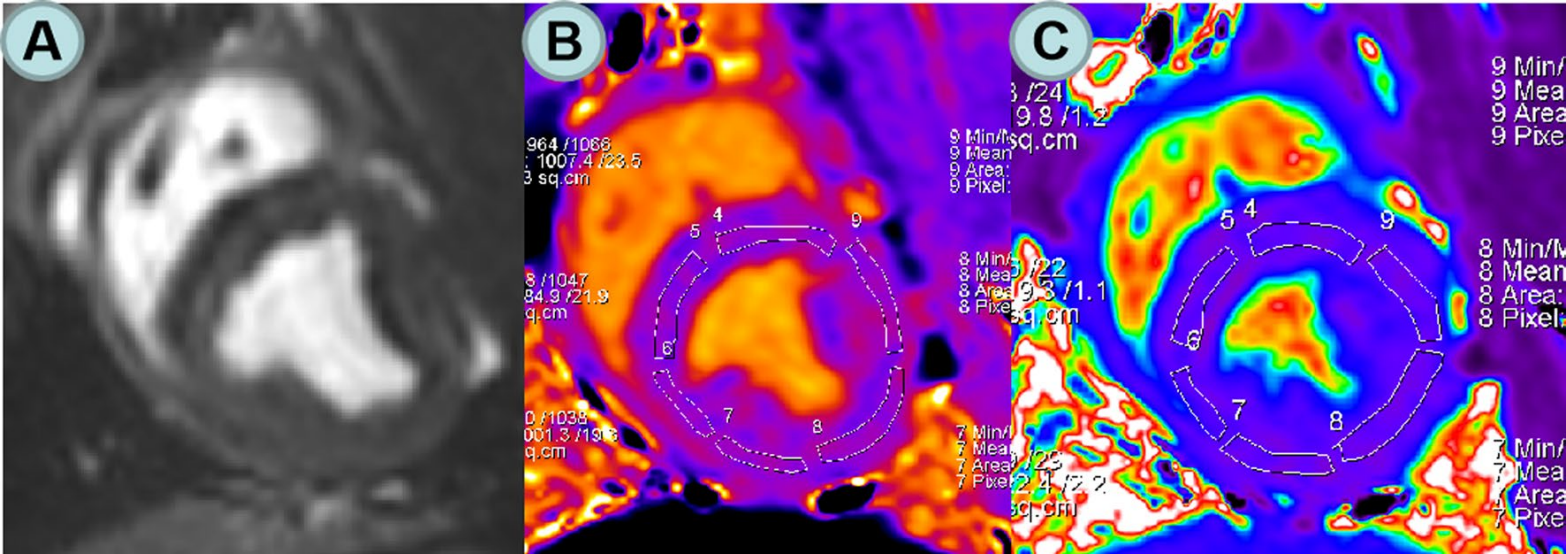
**A** The middle section of the left ventricular short axis was scanned; **B** T1 mapping image in mid short axis views after the mold was made; **C** ECV mapping in mid short axis views after the mold was made

**Fig. 2:**
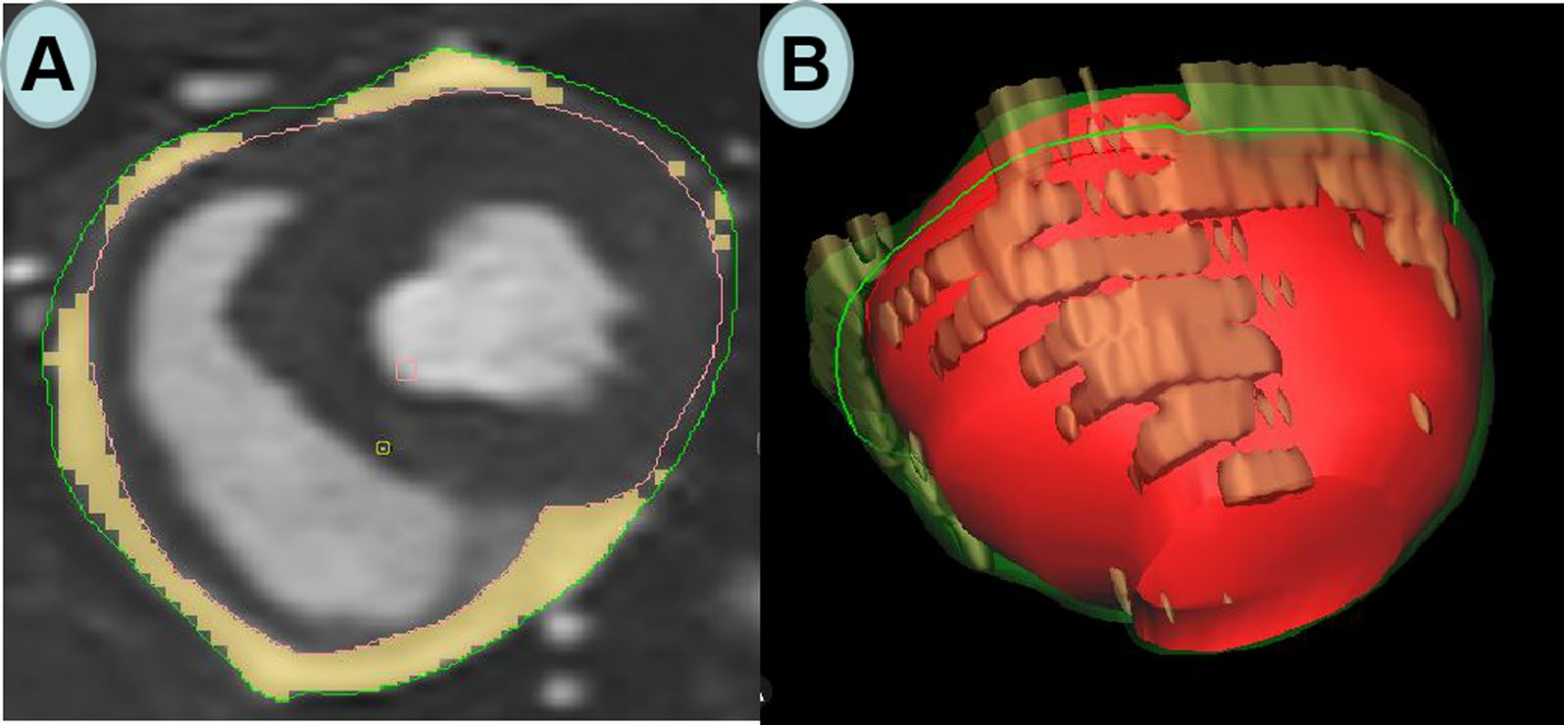
Representative images of epicardial adipose tissue (**A**) and 3D model of epicardial adipose tissue drawn by CVI software (**B**)

Argus software (Syngo MMWP VE30A workstation, Siemens) was used to analyze cardiac structure and function. The basic parameters of cardiac function, including left ventricular end-diastolic volume (LVEDV); Left ventricular end-systolic volume (LVESV);Left ventricular ejection fraction (LVEF); Left ventricular mass (LVM). In order to avoid the influence of partial volume effect, the native T1 and ECV values were measured by manually sketching the region of interest (ROI) in the interventricular septum myocardium of the left ventricular basal, middle and apical segments (Fig. 1). EAT was manually traced on contiguous end-diastolic short axis slices from the base to the apex using a dedicated commercial software (CVi42; Circle Cardiovascular Imaging Inc., Calgary, Alberta, Canada) (Fig. 2). EAT measurement was performed according to the method described by Cai et al. [12].

**Fig. 3:**
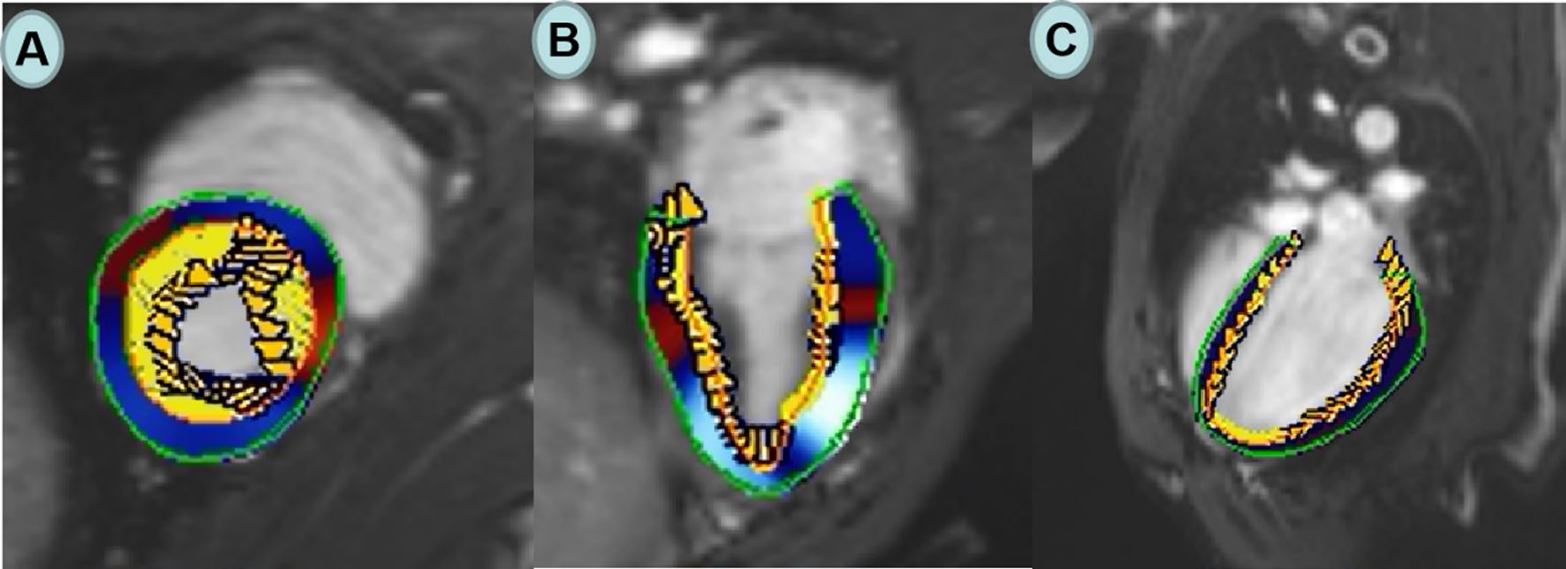
Representative contour of the endocardium and endocardium of the left ventricular in the short axis, 2-, 4-chamber (**A**, **B** and **C**, respectively)

Myocardial systolic strains were analyzed and processed using commercial cardiovascular postprocessing software (Medis 3.0, Netherlands) to obtain global measurements of LV strain. All the continuous short axis films and two-,three-, and four-chamber long-axis images were imported into Medis 3.0 software offline. At the end of diastole, the left ventricular endocardial and epicardial contours were manually delineated on the short axis and the long axis respectively. The trabecular and papillary muscles were included within the heart cavity (Fig. 3A–C). The left ventricle global longitudinal (LVGLS), circumferential (LVGCS) and radial strain (LVGRS) were calculated by automatically tracking the contours in each cardiac cycle.

### Histological examination

At 6 months, after MR scanning, three pigs were sacrificed, and the hearts were collected and fixed in 10% formalin. After dehydration and embedding, sectioning was conducted and the corresponding part was selected in the interventricular septum myocardium, with a slice thickness of 5 μm, followed by Masson stainin.

### Statistical analysis

All data were statistically analyzed using standard statistical software (SPSS 21.0 for Windows, IBM, Chicago, IL, USA). Continuous variable are presented as median and range (lowest and highest). One way analysis of variance (ANOVA) was used to compare baseline and the value of the relevant index at 6 months. For all comparisons, statistical significance was considered at a *p* value < 0.05.

### Reproducibility

The LV strain of three mini-pigs were analyzed independently by two experienced observers. One observer made another analysis after 14 days. The repeatability of parameters within and between observers were calculated respectively. By calculating the inter class correlation coefficient (ICC), the repeatability of multiple measurements was analyzed.

## Results

### MRI characteristics and myocardial strain indexes of diabetic mini-pigs

**Fig. 4:**
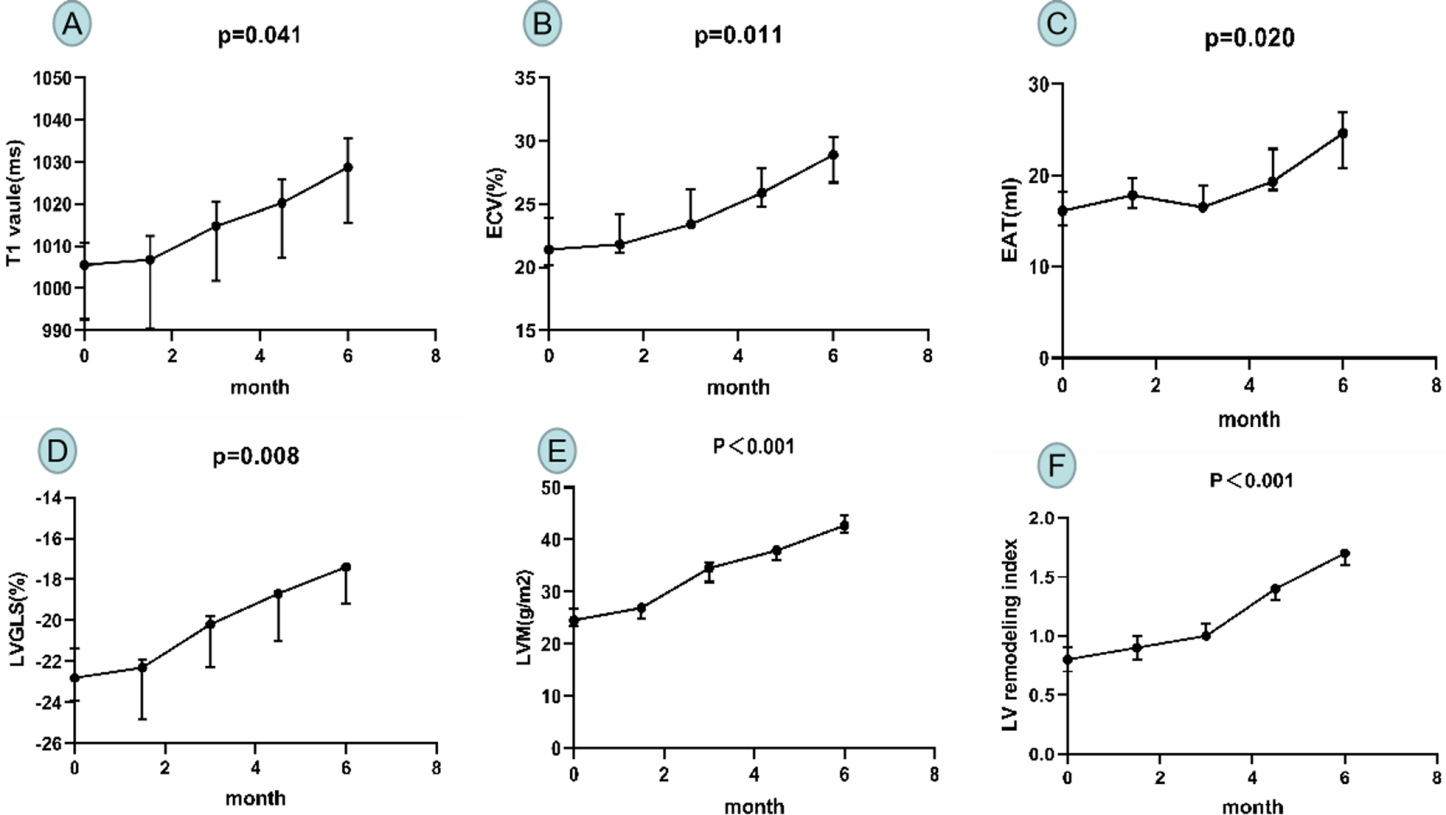
The change trend graph of Native T1 (**A**), ECV (**B**), EAT (**C**), LVGLS (**D**), LVM (**E**) and LV remodeling index (**F**) at different time points. LVM, left ventricular mass; ECV, extracellular volume; GLS, global longitudinal; EAT, epicardial adipose tissue. *P* value for 0 vs 6 month was showed

All the three miniature pigs were successfully modeled, and there were no statistically significant differences in LVEDV, LVESV, LVEF, LVGRS and LVGCS before and after the DCM model (Table1). LVM, LV remodeling index, native T1, ECV value, LVGLS, and EAT were increased at the 6th months of modeling compared to the baseline (LVM: 24.5 (23.4, 26.7) vs. 42.7 (41.4, 44.6) g/m^2^, *p* < 0.001; LV remodeling index: 0.8 (0.7, 0.9) vs. 1.7 (1.6, 1.7), *p* < 0.001; Native T1: 1005.5 (992.6, 1010.7) vs. 1028.7 (1015.5, 1035.6) ms, *p* = 0.041; EAT: 16.1 (14.5, 18.2) vs. 24.6 (20.8, 26.9) mL, *p* = 0.020; ECV: 21.4 (20.2, 23.9) vs. 28.9 (26.7, 30.3) %, *p* = 0.011; LVGLS: − 22.8 (− 21.4, − 23.9) vs. − 17.4 (− 17.2, − 19.2)%, *p* = 0.008) (Table 1, Fig. 4).

**Table 1:** MRI characteristics of the diabetic mini-pigs

Characteristics	Modeling 0 M	Modeling 1.5 M	Modeling 3 M	Modeling 4.5 M	Modeling 6 M	*P* values (0 vs 6 M)
LVEDV (mL/m^2^)	28.1 (25.2, 30.2)	28.7 (26.4, 29.8)	27.9 (27.1, 31.3)	26.4 (26, 32.7)	28.7 (26.5, 30.7)	0.694
LVESV (mL/m^2)^	12.9 (9.1, 16.6)	12.1 (9.7, 15.9)	12.8 (10.1, 15.6)	12.4 (9.5, 17.2)	13.1 (10.2, 16.8)	0.871
LVEF (%)	59.9 (57.2, 64.2)	61.1 (58.3, 66.9)	60.8 (55.6, 68.2)	61.5 (58.6, 66.1)	61.8 (58.4, 67.6)	0.568
LVM (g/m^2^)	24.5 (23.4, 26.7)	26.9 (24.9, 27.3)	34.5 (31.9, 35.6)	37.9 (36.1, 38.5)	42.7 (41.4, 44.6)	< 0.001*
LV remodeling index	0.8 (0.7, 0.9)	0.9 (0.8, 1.0)	1.0 (1.0, 1.1)	1.4 (1.3, 1.4)	1.7 (1.6, 1.7)	< 0.001*
Native T1 (ms)	1005.5 (992.6, 1010.7)	1006.7 (990.5, 1012.4)	1014.7 (1001.8, 1020.5)	1020.2 (1007.3, 1025.8)	1028.7 (1015.5, 1035.6)	0.041*
EAT (ml)	16.1 (14.5, 18.2)	17.8 (16.4, 19.7)	16.5 (16.2, 18.9)	19.3 (18.4, 22.9)	24.6 (20.8, 26.9)	0.020*
ECV (%)	21.4 (20.2, 23.9)	21.8 (21.1, 24.2)	23.4 (23.3, 26.2)	25.9 (24.8, 27.8)	28.9 (26.7, 30.3)	0.011*
LVGRS (%)	57.9 (56.7, 60.9)	62.3 (58.1, 62.3)	59.7 (56.3, 60.3)	60.7 (56.2, 61.2)	58.2 (55.3, 62.0)	1.000
LVGCS (%)	− 22.9 (− 22.3, − 23.8)	− 22.1 (− 20.2, − 23.1)	− 23.9 (− 23.4, − 24.2)	− 22.2 (− 21.2, − 23.3)	− 22.1 (− 20.8, − 24.5)	0.672
LVGLS (%)	− 22.8 (− 21.4, − 23.9)	− 22.3 (− 21.9, − 24.8)	− 20.2 (− 19.8, − 22.3)	− 18.7 (− 18.5, − 21.0)	− 17.4 (− 17.2, − 19.2)	0.008*

LVEDV, left ventricular end-diastolic volume; LVESV, left ventricular end-systolic volume; LVEF, left ventricular ejection fraction; LVM, left ventricular mass; EAT, epicardial adipose tissue; ECV, extracellular volume; LVGRS, left ventricular global radial strain; LVGCS, left ventricular global circumferential strain; LVGLS, left ventricle global longitudinal; LV remodeling index = LV mass/volume ratio; P value for 0 vs 6 month was showed (*P < 0.05)

### Histological analysis of myocardial alterations

**Fig. 5:**
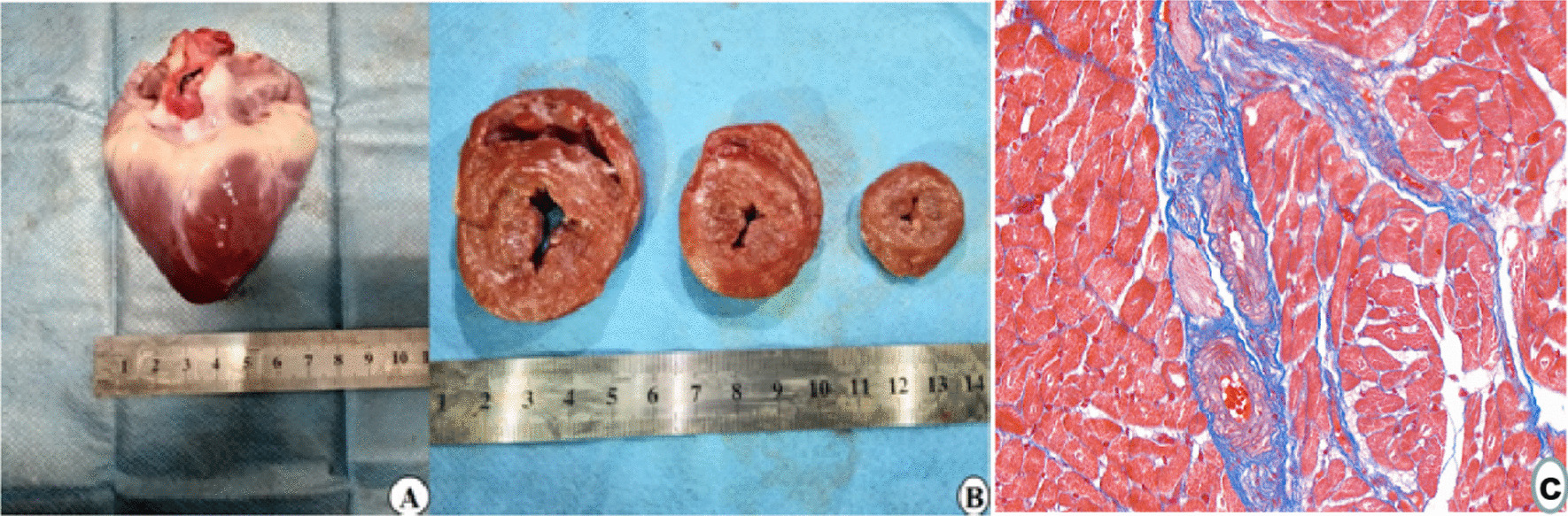
**A** General morphological anatomy of pig heart; **B** Section anatomy in base, mid, apical short axis views were presented; **C** After modeling 6 M, Masson staining of the left ventricular myocardial base segment (blue = fibrosis, red = myocardial fiber)

At the 6th months of modeling, gross anatomy of diabetic model pig heart showed left ventricular wall hypertrophy (Fig. 5A, B), Masson staining of myocardium showed diffuse myocardial interstitial fibrosis (Fig. 5C).

### Repeatability analysis

The intraclass correlation coefficient (ICC) values of nativeT1, ECV, EAT, LVGRS, LVGCS and LVGLS measured by the same physician were 0.974, 0.957, 0.945, 0.985, 0.869 and 0.934 respectively. The ICC values of nativeT1, ECV, EAT, LVGRS, LVGCS and LVGLS were 0.969, 0.973, 0.948, 0.981, 0.893 and 0.926 respectively, measured by two different physicians.

## Discussion

This animal model simulates the type 1.5 DM model of human disease, the advantages of this model not only avoid the effect of various confounding factors in patients on diabetic cardiomyopathy, such as age, the course of the disease, and coexisting diseases, but also obtain the imaging indicators of myocardial fibrosis by non-invasive imaging method and compare them with myocardial histological alterations to insight into the pathogenesis of DCM. The main findings of this study were that (1) the increases of myocardial T1 value, ECV and LVM and the decrease of left ventricular GLS were found at the 3rd months of modeling, and (2) EAT abnormalities were observed at the 6th months of modeling compared to the base line.

T1 mapping can early monitor myocardial focal and diffuse fibrosis by quantifying myocardial T1 and extracellular volume values, and it has been confirmed in previous histological studies [13]. Our results showed that T1 and ECV values of diabetic animal models increased gradually with the prolongation of DM course after 3 months of modeling, which was consistent with the study has been published on T2DM patients [14]. However, the myocardial ECV studied by Sorensen et al. [14] was not confirmed by myocardial biopsy. Due to pigs have many similarities with humans in anatomy, metabolism, physiology and pathophysiology, our findings in histological biopsy samples also lends credence to their findings. There are many factors causing this myocardial damage, including many molecular mechanisms. Animal studies in vivo have shown that hyperglycemia leads to cardiomyocyte apoptosis through the activation of reactive oxygen species (ROS) [15], which in turn can induce the formation of end glycation end products and eventually lead to myocardial fibrosis [16]. These findings also provide the supports for our experimental results. After 3 months’ modeling, T1 value and ECV value gradually increased with the extension of diabetes duration, which was consistent with the results of Zeng [17], indicating that the longer the course of diabetes, the higher the degree of myocardial fibrosis. One possible explanation is that the long term chronic hyperglycemia may aggravate myocardial interstitial matrix expansion, therefore, promoting myocardial apoptosis and necrosis, and eventually, leading to continuous myocardial injury.

In this study, it was found that the LV remodeling index (LV mass/volume ratio) was increased after the modeling of 4.5 months. We also found that LVM was increased after the modeling of 1.5 months. Similar results were reported by Sorensen et al. [18, 19] in a large cohort of patients examined with CMR. In their entire cohort, Sorensen et al. found that LVM was generally higher in these patients with FGF-23 above the median. FGF-23 has been shown to directly affect myocardial cells and induce left ventricular hypertrophy [20]. In our diabetic animal model, the increase in LVM and left ventricular wall hypertrophy in gross specimens further confirmed the previous findings. According to relevant reports, the pathogenesis of diabetic left ventricular hypertrophy was also related to diabetic myocardial interstitial fibrosis and myocardial triglyceride accumulation [21, 22]. In our experiment, diffuse fibrosis of myocardial interstitium has been confirmed in the histological analysis, so next we will expand our sample size, and conduct anti-myocardial fibrosis therapy in diabetic pigs to observe the histological changes of isolated heart to verify whether the above treatment can protect or reverse myocardial damage.

EAT comprises adipocytes, stromo-vascular cells, neurons, and immune cells [23, 24], which is metabolically very active. The secretome of EAT disrupts cardiomyocyte metabolism [25], depresses cardiomyocyte contractile function [5] and alters expression of adhesion markers of primary cardiac endothelial cells [26]. In our study, we also find the EAT in diabetic model was significantly increased at the 6th months diabetic modeling. It's been reported previously that the amount of EAT is increased in patients with T2DM [27] and the high level of EAT is associated with cardiac systolic dysfunction in patients with T2DM [28]. Thus, whether dysfunction of cardiomyocyte contractile function (LVGLS) at the 6th months modeling can be ascribed by the pure effect of DM or by a confounding effect of DM and EAT still requires further study. As the myocardium consists of 3 layers [29], and because the orientation of left ventricle subendocardial muscle fibers is longitudinal, the subendocardial layer is more susceptible to ischemia, toxic, or other metabolic factors [30, 31]. The decrease of LVGLS indicates the subendocardial dysfunction appeared earlier than the global cardiac dysfunction assessed by LVEF in those pigs. Our results show that CMR-FT is a very sensitive tool to detect subtle endocardial impairment in the early stages of DM. In addition, this study also found that the longer the course of diabetes, the lower the value of LVGLS, indicating a progressive damage of the endocardial muscle. Therefore, effective reduction of blood glucose level is essential to protect the myocardium. In the following experiments, we will also conduct hypoglycemic treatment on diabetic pigs to observe whether myocardial mechanical injury can be reversed or ameliorated, so as to provide an important theoretical basis for clinical improvement of cardiac function in patients with DCM.

In this study, three limitations should be considered. First, our animal sample size was small, which greatly reduced the power of the study and did not allow us to draw generalized conclusions. We will continue to expand our sample size in our future studies on this topic. Second, our study was unable to carry out serial histological validation evaluation at various timepoints in type 1.5 DM animal models because pigs are large animals and limited by many experimental conditions. Third, impaired myocardial perfusion is one of the key characteristics of diabetic cardiomyopathy. However, due to technical reasons, we failed to do stress myocardial perfusion after many attempts. In the next experiment, we'll explore this myocardial perfusion imaging technique.

## Conclusions

In summary, our study suggests that increases in LVM, myocardial native T1, ECV, and EAT can be detected prior to a decline in left ventricular systolic function as diabetes progresses in diabetic mini-pigs. LVGLS is impaired gradually with the prolonged course of diabetes. Whether LV systolic dysfunction can be ascribed to the true effect of DM or to a confounding effect of DM and EAT still requires further study. T1 mapping and myocardial strain analysis are promising method in detecting subtle myocardial dysfunction in the early stages of DM.

## Data Availability

The datasets used and analyzed during the current study are available from the corresponding author on reasonable request.

## Abbreviations

DCM: Diabetic cardiomyopathy
LV: Left ventricle
T2DM: Type 2 diabetes mellitus
CMR-FT: Cardiovascular magnetic resonance feature tracking
LVEDV: Left ventricular end-diastolic volume
LVESV: Left ventricular end-systolic volume
LVEF: Left ventricular ejection fraction
LVM: Left ventricular mass
LAV: Left atrial volume
LVGRS: Left ventricular global radial strain
LVGCS: Left ventricular global circumferential strain
LVGLS: Left ventricular global longitudinal strain
EAT: Epicardial adipose tissue
ECV: Extracellular volume
ICC: Intra-class correlation coefficient
